# Pediatric Index of Mortality and PIM2 Scores Have Good Calibration in a Large Cohort of Children from a Developing Country

**DOI:** 10.1155/2014/907871

**Published:** 2014-06-15

**Authors:** Jhuma Sankar, Archana Singh, M. Jeeva Sankar, Sunil Joghee, Shashikant Dewangan, Nandkishore Dubey

**Affiliations:** ^1^Department of Pediatrics, PGIMER, Dr. R.M.L Hospital, New Delhi 110001, India; ^2^Department of Pediatrics, All India Institute of Medical Sciences, New Delhi 110029, India

## Abstract

*Objective*. Our objective was to validate the Pediatric Index of Mortality (PIM) and PIM2 scores in a large cohort of children from a developing country. *Design*. Prospective observational study. *Setting*. Pediatric intensive care unit of a tertiary care teaching hospital. *Patients*. All children aged <18 years admitted between June 2011 and July 2013. *Measurements and Main Results*. We evaluated the discriminative ability and calibration as measured by the area under the receiver operating characteristic (ROC) curves, the Hosmer-Lemeshow goodness-of-fit (GOF), and standardized mortality ratio (SMR), respectively. Of the 819 children enrolled, 232 (28%) died. The median (IQR) age of the study subjects was 4 years (0.8, 10). The major reasons for ICU admission as well as mortality were sepsis/severe sepsis. The area under ROC curves for PIM and PIM2 was 0.72 (95% CI: 0.67–0.75) and 0.74 (95% CI: 0.70–0.78), respectively. The goodness-of-fit test showed a good calibration across deciles of risk for the two scores with *P* values being >0.05. The SMR (95% CI) was 0.99 (0.85–1.15) and 1 (0.85–1.16) for PIM and PIM2, respectively. The calibration across different age and diagnostic subgroups was also good. *Conclusion*. PIM and PIM2 scores had good calibration in our setup.

## 1. Introduction

Scoring systems are used to evaluate the risk of mortality in intensive care units and form an essential part of providing intensive care. They allow for interunit and intraunit comparisons with time and also provide useful information for comparing the severity of illness of patients enrolled into clinical trials [1]. The two commonly used mortality risk scoring systems in children include the Pediatric Risk of Mortality (PRISM) and the Pediatric Index of Mortality (PIM) scores [2, 3]. While both of these scores have been shown to perform well across pediatric intensive care units (PICU), the simplicity of the PIM makes it easier to collect data routinely from large numbers of sick children [4–7]. A number of studies, predominantly from developed countries as well as from a few resource-restricted settings, have validated PIM and its updated version PIM2 scores.

Almost all studies that evaluated the performance of PIM/PIM2 in units from low- and middle-income countries had reported excellent “discrimination” but poor “calibration” of the scores [6–8]. In contrast, in our earlier study on the performance of PIM and PIM2 scores at two different time points involving 282 sick children, we found not only acceptable discrimination but also excellent calibration across the deciles of risk at both of the time points [9]. We were indeed perplexed by this unexpected finding. As opposed to discrimination, which is the ability of a model to distinguish accurately between survivors and nonsurvivors in a given unit, calibration is an actual measure of performance of the scoring system in that unit compared with that of the unit(s) where the original score was developed. Consequently, it may be presumed that the current performance of our unit is quite similar to that of the index units during the time period in which the scores were developed. Given the stark differences in allocation of resources and possibly in the case mix between the index units and our unit, this presumption defied logic. One major factor against this presumption was the possibility of “Type II” error; it is said that the *P* value of Hosmer-Lemeshow GOF test is unreliable with sample sizes of less than 400 [10]. The excellent calibration found in our study could simply be due to the small sample size rather than due to “good” performance of our unit. We therefore undertook this study to evaluate the performance of both PIM and PIM2 scores in a much larger sample of sick children.

## 2. Methods

### 2.1. Design and Setting

We conducted this prospective observational study in our 18-bedded tertiary care PICU from June 2011 to July 2013. Of the 18 beds in the ICU, 10 are in the “intensive area” while the remaining 8 are in the “step down area.” Children aged 1 month to 18 years requiring ICU care from the wards as well as those referred from other hospitals are admitted in the ICU. Our unit caters to both medical and postsurgical patients. Children with traumatic injuries are not admitted in the ICU. The unit is staffed by two full-time pediatric intensivists, 4 fellows, and 4 residents. A total of 14 nurses are posted in the ICU with 4-5 nurses per 8-hour shift. The nurse to patient ratio is 1 : 3 in the intensive area and 1 : 5-6 in the step down area. The ICU is well equipped with facilities for continuous monitoring, mechanical ventilation, blood gas analysis, ultrasonography, and X-ray facilities.

### 2.2. Objectives and Outcome Variables

Our primary objective was to evaluate the discriminative ability and calibration of PIM and PIM2 scores. The secondary objective was to assess the calibration across different age and diagnostic subgroups. The discriminative ability was assessed by the area under the receiver operating characteristic (ROC) curve [11] while calibration was assessed using the Hosmer-Lemeshow GOF test and SMR [12].

### 2.3. Subjects and Data Collection

We included the data from all children admitted to the ICU for more than 1 hour during the study period. Two investigators (AS and SJ) collected the data during the study period. Both of the investigators were trained on the methods of data collection by the principal investigator (JS) at the beginning of the study. The data recorded by them over the next 2 weeks was cross-checked by two investigators (JS and MJS) to ensure correctness; discrepancies, if any, were discussed and resolved. The data collected included all variables of PIM and PIM2, demographic characteristics, clinical course, and outcomes of the study population. Further details on methodology are provided in our previously published article on PIM and PIM2 scores at different time points [9]. Data for 50 children each were recorded in duplicate during the first and the second years of study to ensure accuracy. The interobserver reliability was found to be excellent (*κ* score = 0.93).

### 2.4. Statistical Analysis

Data was entered into Microsoft Excel 2007 (Microsoft Corp., Redmond, CA) and analyzed using Stata 11.2 (StataCorp, College Station, TX). Categorical data are presented as number (%) while continuous variables are presented as mean (SD), if normally distributed, and median (interquartile range), if skewed. Statistical analysis was performed using Student's *t*-test/Wilcoxon rank sum test and chi-square test for continuous and categorical variables, respectively. A *P* value of 0.05 was considered significant.

The performance of PIM and PIM2 scores was assessed by area under ROC curve for discrimination and Hosmer-Lemeshow GOF *P* values and SMR for calibration across deciles of risk, age, and diagnostic subgroups

### 2.5. Ethics and Informed Consent

The protocol was cleared by institutional Ethics Committee. Informed consent was taken from one of the parents of enrolled children.

## 3. Results

**Figure 1:**
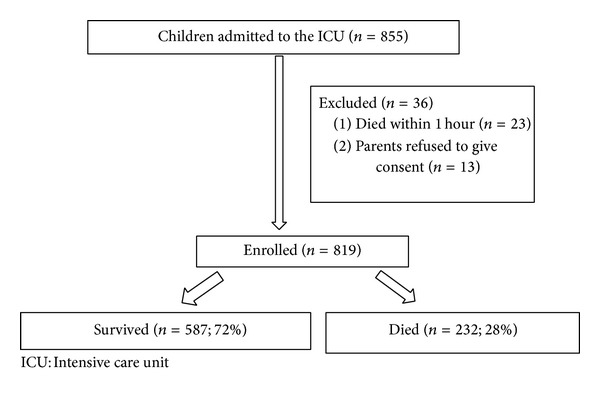
Study flow.

There were a total of 855 admissions during the study period, of which 23 children died within 1 hour and were excluded. Parents of 13 children refused to give consent. The final dataset was comprised of 819 children of whom 232 died (28%) (Figure 1). Data collection of all variables of PIM and PIM2 was possible throughout the study period.

The demographic features, clinical course, and laboratory features of the survivors and the nonsurvivors are provided in Table 1. The median age of the enrolled children was 4 years with the majority being boys (57%). About 28% of the children were less than 1 year of age, 25% were between 1 year and 4 years of age, 22% were between 5 and 10 years of age, and 25% were >10 years of age. About one-fifth (*n* = 164, 20%) of the children were severely malnourished.

The major reasons for ICU admission as well as mortality were sepsis/severe sepsis and cardiac and neurological illnesses. Most of the patients (573, 70%) were admitted directly from the emergency department while the remaining patients were either elective (post-op) or referred from other pediatric wards. The common underlying illnesses in the study population were congenital/structural heart diseases, neurometabolic disorders, and tubercular meningitis (Table 1).

The major cause of death was refractory shock (38%), refractory hypoxemia (25%), raised intracranial pressure and cerebral herniation (15%), and refractory congestive heart failure (22%). The median (IQR) time to death was 5 (3.5, 6) hours.

### 3.1. Primary Outcomes

**Figure 2:**
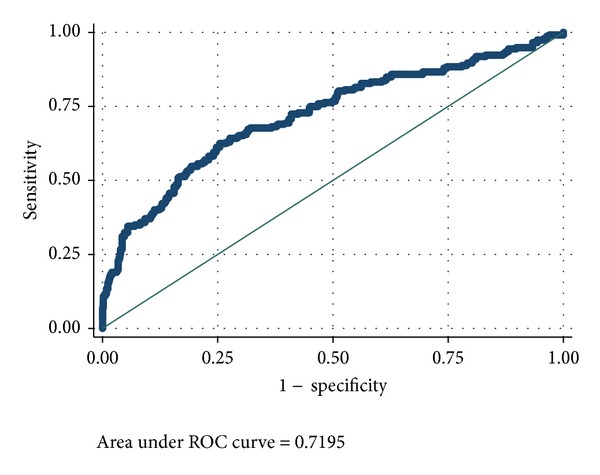
Area under the ROC curve for PIM score. PIM: Pediatric Index of Mortality; ROC: receiver operating characteristic.

**Figure 3:**
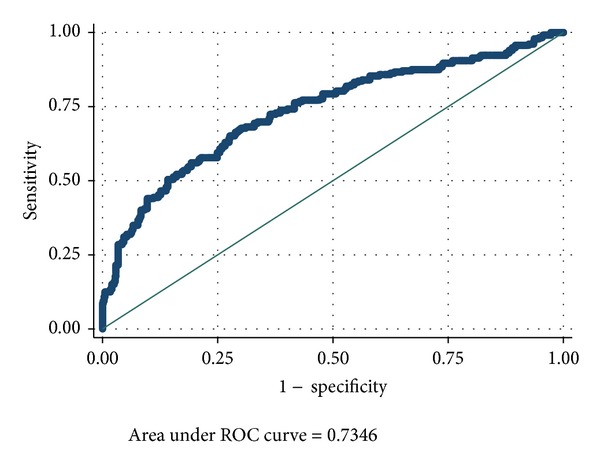
Area under the ROC curve for PIM2 score. PIM2: Pediatric Index of Mortality; ROC: receiver operating characteristic.

Figures 2 and 3 show the area under the ROC curves of PIM and PIM2 scores. The area under the curves (AUC) of PIM and PIM2 scores was 0.72 (95% CI: 0.67–0.75) (Figure 2) and 0.74 (95% CI: 0.70–0.78) (Figure 3), respectively. Both scores showed good calibration across deciles of risk as measured by the GOF test with Hosmer-Lemeshow *P* > 0.05 (Table 2). Calibration as measured by SMR was also good for the two scores. The number of deaths predicted by PIM was 232.1 (SMR 1; 95% CI: 0.88–1.13) and by PIM2 was 232 (SMR 0.99; 95% CI: 0.87–1.12). A closer look across each decile of risk showed that both PIM and PIM2 scores underpredicted deaths in groups with probability <13%. In addition, PIM underpredicted deaths between 30 and 40%, while PIM2 underpredicted deaths in groups with probability between 40 and 57%. Overprediction was evident in the risk groups of 15–17% for both PIM and PIM2 scores (Table 2).

### 3.2. Secondary Outcomes

Calibration across different age and diagnostic subgroups was also good with GOF *P* values being >0.05 across most of the subgroups for both PIM and PIM2 scores (Table 3). Individually, PIM2 score had good calibration across all age categories in comparison to PIM score which had poor calibration among children in the 2–5-year age group. Between the diagnostic subgroups, calibration was poor only in postoperative patients for the two scores. Discrimination was best for respiratory illnesses, poisoning, liver failure, and tubercular meningitis for the two scores (Table 3).

## 4. Discussion

The results of the present study confirm our earlier findings of excellent calibration but acceptable discriminatory performance of both PIM and PIM2 scores in our setup. The numbers of predicted and observed deaths were almost equal for both scores across deciles of risk, age, and diagnostic subgroups. The results of our previous study were therefore not merely due to chance. As previously mentioned, calibration is an important measure of validation of a scoring system in a unit in which it was not developed. The measure of calibration—SMR—is basically a comparison of the number of deaths predicted by the scoring system with the number of observed deaths. According to the investigators of the original PIM and PIM2 scores, the SMR that is significantly different from 1 in a given unit means that the standard of care in that unit is worse (or better, depending on the direction) than the units that derived the score [10]. It is but natural to expect that the observed deaths in a given unit would be similar to the number of expected deaths so that the SMR equals 1. However, this is often not true, and, depending on the case mix and disease patterns, the SMR might vary and may be significantly different from 1 (i.e., 95% CI of SMR would not include 1); in these cases, the Hosmer-Lemeshow *P* values would be less than 0.05.

The results of our study are different from most other studies from developing countries that reported the models to be underpredicting the deaths in their setup, with the SMR and its 95% CI being more than 1 [6–8]. For example, two studies from India and Pakistan have reported SMRs as high as 1.57 to 3.3 and 1.4 to 1.57 for PIM and PIM2 scores, respectively [7, 8]. The study authors have attributed this to the differences in the patient profile, need to manage large numbers of severely ill children with limited manpower and resources, and possible differences in quality of care between their units and the units where the models where developed. In contrast, we found the SMR to be equal to 1 not only across deciles of risk but also across all age and diagnostic subgroups. We presume that factors like the threshold for initiating and discontinuing support, timing of intensive care admissions, and quality of care as well as the accuracy of data collection might have contributed to the near-perfect SMR in our unit. It appears that resource limitation may not be a major deterrent to imparting quality care in the ICU. It may be more important to review the systems in place and take steps to improve them in order that the performance of the models improves in units where the models underpredict deaths.

Unlike the units in which the scores were developed [3, 13] and most other units from developed countries [5, 14–17], we found only acceptable discrimination of the scores in our setup. The possible reasons for this difference are the high mortality rates in our study (28%) as compared to the units in which the scores were developed or validated (5-6%) [3, 13] and the difference in disease patterns between these units and our unit. For example, we had more children with sepsis, cardiac and neurological illnesses, and raised intracranial pressure. These factors could not be accounted for by the variables used to calculate the scores. Moreover, the case mix and the severity of illness at admission resulted in regression coefficients that are quite different between the development set and our study for some of the items of the scores (Table 4). For example, the coefficient of the item “elective admission” in PIM score was less than half of the original dataset. This is possibly due to the fact that almost all admissions in our setup are emergency in comparison to the development sets where almost 50% of the admissions are elective. Similarly the variable “cardiac bypass” was omitted from the PIM2 model as there were no patients admitted after such procedure in our unit. When it came to the variables of high risk and low risk diagnosis there were major differences between the development set and our setup with regard to these. For example, poisoning has a low risk of mortality in our setup and this could not be accounted for in the PIM2 “low risk diagnosis" variable as it does not have poisoning among its low risk diagnoses category. Among the cardiac illnesses, only dilated cardiomyopathy or myocarditis is included in the “high risk diagnoses category" of both PIM models. In our unit, only 2 of the 10 children with an admission diagnosis of acute myocarditis died. Despite these differences, we did not try to improve on the fit of the model by changing the coefficients as this defeats the main purpose of these models which is to allow for interunit comparisons [3, 10, 13]. A few studies from the developed countries did report only acceptable discrimination for PIM and PRISM [4, 18]. One of these studies attributed the poor discrimination to differences in patient demographics and physiologic response to different diseases [18].

The dichotomy between discrimination and calibration that we observed in our study has been previously reported in only a few studies. A study from Trinidad reported an AUC for PIM2 of only 0.62 while the SMR was 0.86 with the 95% CI including 1 [19]. The authors attributed this to overprediction of mortality in their study, but it could mean that their unit performed better than the development sets. Similarly, a study of 303 patients from the Netherlands reported an AUC of 0.74 for PIM score and an SMR of 0.88 with the 95% CI including 1 [4]. It is often said that a perfectly calibrated model may not always be perfectly discriminatory as the area under the ROC curve would be 0.83 and not 1 in such cases [20].

*Strengths and Limitations*. The strengths of our study are: (a) it is the largest study till date from developing countries to validate the PIM and PIM2 scores, (b) data were collected accurately, and (c) the scores were calibrated well in our setup with an adequate sample size, thereby meaning that the scores could be used in units with resource limitation as such without any modifications. The only limitation is that it is a single-unit study. However, this fact is unlikely to affect the generalization of our results as our unit is fairly representative of most units form developing countries with high incidence of sepsis, tuberculosis, and meningoencephalitis cases.

## 5. Conclusion

Contrary to most previously published studies from developing country settings, PIM and PIM2 scores had good calibration in our setup. The good calibration was despite the differences in case mix and resource allocation between the units where the scores were developed and ours.

## Figures and Tables

**Table 1:** Baseline characteristics of study patients.

Variables	All patients (*n* = 819)	Discharged (*n* = 587)	Died (*n* = 232)	*P* value
Median age in years	4 (0.8, 10)	3.5 (1, 10)	3 (0.7, 10)	0.47
Boys	467 (57)	352 (60)	95 (57)	0.57
Elective admission	30 (3.7)	22 (3.7)	8 (3.4)	0.93
Severe PEM	164 (20)	111 (19)	49 (21)	0.23
Diagnostic subgroups (admission)				**<0.001**
Sepsis/severe sepsis	377 (46)	282 (48)	95 (41)	0.02
Cardiogenic shock/CHF/arrhythmia	98 (18)	36 (6)	62 (26)	0.8
Status epilepticus/raised ICP (noninfective)	82 (10)	31 (5)	51 (22)	0.08
Respiratory (noninfective)	49 (6)	47 (8)	2 (0.8)	**0.004**
Postoperative patients	28 (3.4)	27 (5)	1 (0.6)	0.67
Poisoning	98 (12)	97 (17)	1 (0.6)	**<0.001**
Liver failure	25 (3)	13 (2)	12 (5)	0.27
Other conditions (aplastic anemia/DKA/hypertension/uremic encephalopathy)	62 (7.5)	54 (9)	8 (3)	0.06
High risk diagnosis (PIM)	74 (9)	41 (7)	33 (14)	0.016
High risk diagnosis (PIM2)	73 (8.9)	42 (7.1)	31 (13)	0.01
Low risk diagnosis (PIM2)	44 (5.4)	40 (6.8)	4 (1.7)	0.01
Any underlying chronic illness	311 (38)	200 (34)	111 (48)	<0.001
Chronic kidney disease	29 (3.6)	24 (4)	5 (2)	0.46
Congenital/structural heart disease	123 (15)	65 (11)	58 (25)	0.21
Chronic liver disease	4 (0.5)	1 (0.1)	3 (1.3)	0.21
Nephrotic syndrome	49 (6)	47 (8)	2 (0.8)	0.07
Neurometabolic disorder/birth asphyxia/developmental delay	41 (5)	23 (4)	18 (7.7)	0.07
Tubercular meningitis	25 (3)	12 (2)	13 (5.6)	0.01
Malignancies/aplastic anemia/immunodeficiencies	40 (5)	28 (5)	18 (8)	0.1
Hospital course				
Need for MV during 1st hour	180 (22)	78 (13)	102 (44)	<0.0001
Need for blood products	368 (45)	200 (34)	168 (72)	<0.0001
Median duration of inotropes (hrs.)	48 (30, 96)	72 (48, 96)	48 (24, 72)	0.005
Median duration of ventilation (hrs.)	48 (24, 76)	52 (39, 96)	48 ( 23, 72)	0.16
Median duration of ICU stay (days)	5 (2, 7)	4 (2, 6)	5 (3, 7)	0.02

**Table 2:** Calibration across deciles of risk and standardized mortality ratios for the two scores.

Model	Probability of death across 10 risk groups	Deaths across deciles of risk		Hosmer-Lemeshow goodness-of-fit test		Standardized Mortality Ratio (95% CI)
		Observed	Expected	Chi-square	*P* value	
PIM				11.1	0.20	1 (0.88–1.13)
Group 1	0.14	15	9.4			
Group 2	0.16	14	12			
Group 3	0.17	6	13.4			
Group 4	0.19	13	14.8			
Group 5	0.21	18	16.4			
Group 6	0.24	16	18.6			
Group 7	0.30	23	22.2			
Group 8	0.40	33	29.2			
Group 9	0.56	36	38.3			
Group 10	0.96	58	57.8			
PIM 2				12	0.16	0.99 (0.87–1.12)
Group 1	0.13	14	8.3			
Group 2	0.15	10	11.7			
Group 3	0.17	7	12.8			
Group 4	0.18	13	14.3			
Group 5	0.21	15	15.9			
Group 6	0.25	17	18.8			
Group 7	0.32	27	22.9			
Group 8	0.40	27	29.4			
Group 9	0.58	45	38.4			
Group 10	0.94	57	59.4			

PIM: Pediatric Index of Mortality; CI: confidence interval.

**Table 3:** Calibration of the scores across age and diagnostic subgroups.

Variable	*N* = 819	PIM		PIM2	
		Area under ROC curve	Hosmer-Lemeshow chi-square (*P* value)	Area under ROC curve	Hosmer-Lemeshow chi-square (*P* value)
Age range					
<1 year	229 (28)	0.64	9.8 (0.27)	0.67	10 (0.2)
1–4 years	205 (25)	0.80	11 (0.06)	0.79	8.3 (0.4)
5–10 years	180 (22)	0.74	10.45 (0.23)	0.79	3.9 (0.8)
≥10 year	205 (25)	0.75	6.9 (0.5)	0.75	7.2 (0.5)
Diagnoses					
Sepsis	377 (46)	0.73	6.3 (0.6)	0.74	7.7 (0.45)
Cardiac	98 (18)	0.62	0.38 (8.5)	0.70	4.4 (0.8)
Neurological	82 (10)	0.73	0.19 (11)	0.75	5.6 (0.7)
Respiratory	49 (6)	—	—	0.97	0.45 (1)
Postoperative	28 (3.4)	—	—	0.68	13 (0.05)
Poisoning	98 (12)	—	—	—	—
Liver failure	25 (3)	0.70	6.9 (0.5)	0.85	10 (0.22)
Other conditions	62 (7.5)	0.60	9 (0.33)	0.5	8.2 (0.4)

**Table 4:** Logistic regression model of PIM and PIM2 in the study sample.

	Coefficient (95% CI) in our study	*P* value	Coefficient (95% CI) in development set	*P* value
Variables: PIM				
Pupils fixed to light (yes/no)	2.38 (1.32, 3.44)	0.004	2.35 (1.87, 2.84)	<0.00005
Specified diagnosis (yes/no)	**0.73** (0.04, 1.42)	0.037	1.82 (1.45, 2.2)	<0.00005
Elective admission (yes/no)	**−0.67** (−1.74, 0.39)	0.21	−1.55 (−1.94, −1.16)	<0.00005
Mechanical ventilation (yes/no)	1.72 (1.24, 2.19)	<0.001	1.34 (0.95, 1.72)	<0.00005
Absolute (SBP-120) mm Hg	**0.19** (0.006, 0.33)	0.004	0.021 (0.014, 0.027)	<0.00005
Absolute base excess (mmol/l)	**0.004** (0.03, 0.04)	0.845	0.071 (0.046, 0.095)	<0.00005
100× FiO_2_/PaO_2_ (mm Hg^−1^)	**0.89** (0.28, 1.49)	0.004	**0.41** (0.23, 0.59)	<0.00005
Constant	−2.48 (−3.01, −1.9)		−4.873 (−5.25, −4.49)	
Variables: PIM 2				
Pupils fixed to light (yes/no)	2.67 (1.51, 3.8)	0.008	3.07 (2.77, 3.38)	<0.0005
Elective admission (yes/no)	Omitted	—	−0.92 (−1.17, −0.6)	<0.0005
Mechanical ventilation (yes/no)	1.89 (1.41, 2.37)	<0.001	1.37 (1.08, 1.65)	<0.0005
Absolute (SBP-120) mm Hg	0.01 (0.005, 0.03)	0.008	0.01 (0.013, 0.017)	<0.0005
Absolute (base excess) mmol/l	0.006 (0.03, 0.04)	0.77	0.10 (0.09, 0.11)	<0.0005
100× FiO_2_/PaO_2_ (mm Hg^−1^)	0.97 (0.34, 1.6)	0.002	0.28 (0.20, 0.37)	<0.0005
Recovery post procedure (yes/no)	0.98 (−2.1, 0.1)	0.09	−0.93 (−1.2, −0.6)	<0.0005
By pass (yes/no)	Omitted		0.75 (0.39, 1.10)	<0.0005
High risk diagnosis (yes/no)	0.78 (0.10, 1.4)	0.023	1.68 (1.5, 1.8)	<0.0005
Low risk diagnosis (yes/no)	−0.99 (−2.2, 0.29)	0.13	−1.57 (−2.02, −1.12)	<0.0005
Constant	−2.55 (−3.1, −1.9)		−4.88 (−5.11, −4.6)	

SBP: systolic blood pressure; FiO_2_: fraction of inspired oxygen; PaO_2_: partial pressure of oxygen; PIM: Pediatric Index of Mortality; CI: confidence interval.
